# Sending money home: a mixed-Methods study of remittances by migrant nurses in Ireland

**DOI:** 10.1186/1478-4491-7-66

**Published:** 2009-07-30

**Authors:** Niamh Humphries, Ruairí Brugha, Hannah McGee

**Affiliations:** 1 Division of Population Health Sciences, Royal College of Surgeons in Ireland, Dublin, Ireland

## Abstract

**Background:**

This paper presents data on the remittances sent by migrant nurses to their families "back home". It gives voice to the experiences of migrant nurses and illustrates the financial obligations they maintain while working overseas. Although the international economic recession has decreased global remittance flows, they remain resilient. Drawing on the experiences of migrant nurses in Ireland, this paper indicates how and why migrants strive to maintain remittance flows, even in an economic downturn.

**Methods:**

A mixed-methods approach was employed, and the paper draws on data from qualitative in-depth interviews undertaken with 21 migrant nurses in addition to a quantitative survey of 336 migrant nurses in Ireland.

**Results:**

The survey of migrant nurses revealed that 87% (293) of the sample sent remittances on a regular basis. According to respondents, remittances made a huge difference in the lives of their family members back home. Remittances were used to ensure that family members could obtain access to health and education services. They were also used to provide an income source for family members who were unemployed or retired.

As remittances played an essential role in supporting family members back home, respondent migrant nurses were reluctant to reduce the level of their remittances, despite the onset of a global recession. Respondents noted that an increased demand for remittances from their families coincided with a reduction in their own net salaries – as a result of increased taxes and reduced availability of overtime – and this was a cause for concern for Ireland's migrant nurses.

**Conclusion:**

This paper provides insights into the importance of remittances in funding social support for family members in home countries. It also illustrates the sacrifices made by migrant nurses to ensure continuation of the remittances, particularly in the context of an economic recession.

## Background

"As millions migrate north, billions flow south" [1]. This paper is about remittances: the money sent by emigrants to their families "back home". Remittance flows are key to understanding how the lives of those who migrate and those who remain at home are altered by migration [2]. The remittance trail connects destination countries with the source countries from which migrant nurses have been recruited, and reminds us of the vast "disparities in economic and professional opportunities" [3] that exist between them.

The necessity of supplementing family incomes provides migrants with a powerful incentive to migrate. In that sense, remittances are a cause as well as an effect of migration [4]. As Harding notes: "Sustaining the remittance, rolling access to foreign income across two generations ... these are powerful motives for migrants" [5].

The "transfer home of migrant earnings and savings is generally seen as the most important positive effect of migration for the countries of origin" [6]. Yet the money itself is just the starting point in analysing the significance of remittance flows:

"Remittances represent far more than simple financial transactions; they are the outcome of the separation of families, the disruption of national economies and the exodus of creative and hardworking adults from poor to richer countries. These flows deliver high financial benefits – but at a very high human cost" [7].

The social cost to migrant workers and their families can be significant, as Parreñas illustrates: "Instead of the father routinely arriving home to his family at supper time, he comes back from work every ten months" [8]. A UNICEF study "estimates that one in four children in the Philippines has at least one parent employed abroad" [9]. Despite the disruption to family life that results from migration, the "commitment to family" [10] remains central to the decision to migrate (and to remit) and "in this sense, remittances can be truly characterised as the human face of globalisation" [10].

This paper gives voice to the experiences of migrant nurses, drawing on qualitative and quantitative data to illustrate the remittance connections maintained while living and working in Ireland.

## Methods

Ethical approval for the Nurse Migration Project was granted by the Research Ethics Committee of the Royal College of Surgeons in Ireland. The study, funded by the Irish Health Research Board 2006–2009, applied both qualitative and quantitative methods to the study of migrant nurses in Ireland. The mixed-methods approach was invaluable to the study, adding breadth and depth to the analysis [11] and helping to ensure the comprehensiveness of the data [12].

Although this paper focuses on remittances, this was just one of several issues explored with respondent migrant nurses in both interviews and questionnaires. Remittances were initially discussed with respondent migrant nurses during in-depth interviews; the level and scope of financial support provided to the wider family came as a surprise to the research team. Following on from that, five remittance-related questions were incorporated into the survey questionnaire in order to ascertain whether those experiences were typical of the migrant nurse experience more generally.

### Qualitative Methods

The initial fieldwork phase involved qualitative methods. In-depth interviews were conducted with 21 migrant nurses working in Ireland in 2007. Accessing a sample of migrant nurses proved a difficult task.

The Irish Nurses Organisation (INO), Ireland's largest professional union for nurses and midwives [13], was approached to assist in the recruitment of migrant nurse research participants. The INO Overseas Nurses Section[13,14] has a membership of approximately 5000 identifiable migrant nurses. A campaign of industrial action by the INO immediately prior to the fieldwork phase [15] served to boost union membership but nevertheless the INO represents, at best, 5000 of the 9441 non-European Union nurses issued with working visas between 2000 and 2006 (Irish Department of Enterprise Trade and Employment, unpublished data).

The INO agreed to forward letters on behalf of the research team to a randomly selected sample of 250 of its migrant nurse membership. However, this approach resulted in the recruitment of only eight respondents (Humphries, Brugha, McGee: 'I won't be staying here for long': A qualitative study on the retention of migrant nurses in Ireland, submitted). The recruitment process proceeded by placing articles in migrant newspapers and via snowball sampling: a process of chain referral whereby respondents and gatekeepers are used to refer the researcher to other potential respondents [16].

A sample of 21 migrant nurses resulted (19 women and two men). Most came from the Philippines (16) and India (4); one nurse came from Nigeria. In terms of marital and family status, the majority (17) of respondents had children; most respondents were married (15), three were single, two were separated and one was widowed.

Interviews were conducted in non-workplace settings to facilitate a free and open discussion of experiences. Interviews lasted an average of 69 minutes, beginning with a discussion of confidentiality wherein respondents were invited to select a pseudonym to ensure the anonymity of their responses in various research outputs. Interviews progressed to cover topics such as the decision to migrate, the recruitment process, orientation and adaptation programmes, nursing and living in Ireland and future plans. Interviews concluded with an exploration of topics considered more sensitive, such as remittances and the ethical issues raised by overseas nurse recruitment.

On completion of the interview, all respondents were presented with a modest gift voucher to thank them for their participation and to cover any costs incurred [17]. Interviews were audio recorded and were later transcribed verbatim.

Analysis of qualitative data was undertaken on an ongoing basis throughout the data collection [18] and transcription phases, as the researcher (NH) familiarized herself with emerging research themes. Further inductive analysis was conducted via a thorough re-reading of interview transcripts [19]. Data management and analysis were facilitated by the use of the MaxQDA computer package.

### Quantitative Methods

A quantitative survey of migrant nurses was conducted in early 2009, informed by the qualitative fieldwork undertaken in 2007. The survey contained questions relating to respondents' nursing skills, qualifications and grade prior to migration, the recruitment process, immigration status, arrival, adaptation and orientation, various nursing jobs held in Ireland, career opportunities, experiences of bullying, remittances and future plans. The questionnaire was reviewed by a migrant nurse key informant prior to its circulation and minor modifications were made as a result of feedback received.

In order to gain access to a random sample of migrant nurses in Ireland, the researchers approached the Irish Nursing Board. Registration with the Irish Nursing Board is mandatory for those wishing to practise nursing in Ireland [20]. On behalf of the research team, the Irish Nursing Board forwarded self-completion postal surveys to a random sample of 1536 non-European Union migrant nurses. Respondents were asked to return the questionnaires by post to the research team; a prepaid envelope was provided for the purpose.

In addition to the provision of a prepaid envelope for the return of surveys, a number of measures were employed in an attempt to maximize the survey response rate [21]. First, a postcard was forwarded to each of the 1536 potential respondents in advance of the survey, introducing the research and informing them of the imminent arrival of the questionnaire. Incentives were also used: all those who completed the survey were invited to take part in a drawing for one of three EUR 500 travel vouchers; a small donation to charity was also made for every completed survey received.

A low response rate of 25% was anticipated, in line with previous migrant surveys in the Irish context [22]. Thus a sample size of 384 was sought to enable a +/- 5% margin of error based on an overall migrant nurse population of approximately 11 288 (Irish Department of Enterprise, Trade and Employment, unpublished data) (no precise figure for the number of migrant nurses in Ireland is available, since although immigration of nurses is measured, emigration is not). The postal survey achieved a response rate of 20%; a sample size of 308 was achieved.

A parallel sampling strategy, involving the recruitment of migrant nurses via their hospital employers, was also employed. Three large hospitals in the Dublin area were selected as research sites; ethics approval was sought and received from each institution. In each hospital, recruitment was facilitated by the Nursing Administration Department, whose staff circulated postcards and posters advertising the research project on behalf of the research team. Migrant nurses were invited to meet the researcher on-site at a specified time and date and to participate in the research project by completing a self-completion questionnaire. Surveys were returned to the research team by post; a prepaid envelope was attached to each survey for this purpose. This recruitment strategy also yielded a low response rate, with only 28 non-European Union nurses recruited in this manner. Quantitative data (N = 336) were input and analysed in SPSS software; the analysis of open-ended survey responses was facilitated by the use of MaxQDA software.

The recruitment process resulted in a sample of 336 migrant nurses, of whom 85% were women. Most nurses who completed the survey originated from the Philippines (52%) or India (33%), with the remainder from 14 other countries – including 2% to 3% each from Australia, South Africa, the United States of America and Zimbabwe. The nationalities represented in the sample were broadly similar to those recorded in immigration data (Irish Department of Enterprise, Trade and Employment, unpublished data), although the sample overrepresented Filipino nurses, who accounted for 52% of respondents but 45% of non-European Union nurses who were issued visas. The sample also underrepresented Indian nurses, who accounted for 33% of respondents but 45% of non-European Union nurses who were issued visas (Irish Department of Enterprise, Trade and Employment, unpublished data).

Most of those surveyed (40%) arrived between 2000 and 2002, with a further 29% arriving in 2005–2006. Once again, this is broadly in line with immigration data, which indicate that 35% of migrant nurse visas were issued between 2000 and 2002 and another 35% were issued in 2005–2006 (Irish Department of Enterprise, Trade and Employment, unpublished data). Due to the lack of additional data on the general migrant nurse population in Ireland, no further cohort comparisons can be made. However, in terms of an age profile of the sample population, 30% of respondents were aged 36–40 and a further 26% were aged between 31 and 35. The majority (77%) were married; 68% had children. In terms of nursing experience, 39% of respondents had 6 to 10 years of nursing experience upon arrival.

This paper draws on both qualitative and quantitative findings throughout. Where open-ended survey responses appear, they are referenced according to the number assigned to the questionnaire during data input, whereas qualitative findings are attributed to respondents via their pseudonyms.

## Results

### Pressure to remit

**Figure 1:**
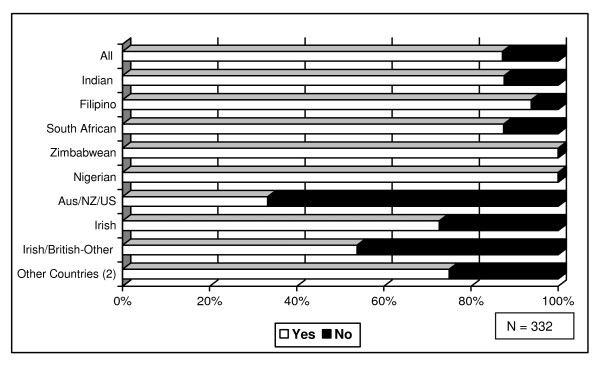
**Percentage sending home remittances, by nationality**.

Ireland's migrant nurses originate primarily from India and the Philippines [23]. Just as the need to remit was a factor in the decision to migrate to Ireland, remittances remained high on their agenda once here, as respondents remained ever-conscious of the need to remit to support family members "back home" [24,25]. Eighty-seven percent (293/336) of the migrant nurses surveyed reported that they sent remittances back home (Figure 1).

The exceptions to these remittance trends were nurses from Australia/New Zealand and the United States, who, as would be expected, were less likely to remit. Those respondents who had acquired Irish or another European Union citizenship were also slightly less inclined to remit: 63% (15/24) of such respondents sent remittances. This reduction in remittance flow could relate to the length of time in-country, as the acquisition of citizenship takes approximately 10 years in the Irish context, although the numbers involved are small.

Although respondents were glad to be able to help family members, there was no doubting the pressure it placed them under:

"You don't want to lose the job, we have a family back home ... like me, I have a mother ... who's sick as well back home, who's awaiting for my salary every month to send her ... so we can't afford to lose our job being here" (Fatima).

In the Philippines in particular, such pressure (both to migrate and to remit) is commonplace, as between 34% and 54% of the Filipino population is sustained economically by migrant remittances [26]. In 2000, the Government of the Philippines appealed to Filipinos overseas to remit more to help stem the depreciation of the peso [27]. Other developing countries and regions are also heavily reliant on remittance income. For instance, remittances contribute around one sixth of Albania's gross domestic product (GDP) [2]; in Kerala, whence many Indian migrant nurses in Ireland were recruited, remittances make up 10% of GDP [6,28].

Research has found that nurses are particularly good remitters and are more likely than other migrants to send remittances home [24,29]. These studies suggest a number of reasons for the "higher remittance propensity among nurses" [24], including the fact that women tend to be more frequent and generous remitters than men and also that nurses, as members of a caring profession, may be more responsive to the needs of their wider families [24]. A further suggestion from Brown and Connell is that "where a migrant's investment in human capital and choice of occupation was driven mainly by prospects for migration, they were likely to be more generous and reliable remitters" [29].

Whatever the motivation for remitting, it would appear that the financial burden of remittances occasionally proved too great and that migrant nurses overstretched themselves financially in Ireland in order to send home that much-needed assistance:

"Sometimes maybe people are tempted to get loans because the banks are the ones who are asking you to ask for loans and then families in the Philippines are asking more money as well" (Carlo).

Companies such as Western Union, whose business it is to facilitate remittance transfers, are quick to reinforce the pressure and to keep remittances in the forefront of migrants' minds through the use of emotive advertising campaigns ("Can love be transferred? Yes": Western Union advertisement campaign, March 2009). Of those survey respondents who sent remittances home (N = 293), 35% (102) reported that they did not struggle financially as a result, while 65% (191) reported that they struggled, at least occasionally, as a result of their remittance commitments. In-depth discussions with migrant nurses revealed that they made considerable sacrifices to ensure the continuation of the remittance flow. Their willingness to do this is an indication of the extent to which extended families relied upon their remittance.

Kingma noted that, although voluntary migrants, some nurses have little choice but to emigrate [30]; this is echoed by Brown, who noted that the Jamaican nurses in his study had been "forced by the economic crisis" [31] to migrate. In this context, migration is used as a "life change strategy" [32] to secure financial survival [33] and/or provide greater financial security for the wider family. Migration may also improve the individual nurses' social standing back home [33,34]. Our in-depth interviews with migrant nurses indicated that similar reasons meant that respondents sometimes had little choice but to remain overseas:

"So we are only forced to stay because financially we're okay ... we are forced, because we need the money, we have to send some to the Philippines" (Agatha).

The pressure to remit also caused some respondent migrant nurses to curtail their career plans and others to remain in jobs in which they were unhappy. It appeared that any action that posed a risk – however temporarily – to the remittance flow was avoided, regardless of the personal cost. These findings corroborate the findings of a Royal College of Nursing study which found that internationally recruited nurses were more likely to work rotating shifts and to work overtime than United Kingdom-trained nurses [35]. Ensuring that the remittance flow was maintained was a priority for respondent migrant nurses:

"It would really take a lot of money to go to school and I can't afford that at the moment because I'm sending money home" (Fatima).

"In my first few months, I really wanted to go home ... but then, still keep on going because we came here in Ireland [for] better compensation, a better way of living. But then, on the counter-part, it's just like our heart is kind of crying" (Mary).

The nurses themselves did not appear to consider these actions as a sacrifice, nor their remittances as a burden, although to an outsider their actions appear extraordinarily generous. As one respondent explained, Irish people simply don't understand the obligation to remit:

'My sister who is unemployed with the children and granddaughter with her and most of their expenses comes from me, now nobody will understand that in an Irish point of view' (Lorna).

For those of us living in a wealthy destination country, it may be difficult to fathom a situation in which State support for the vulnerable in society is minimal or nonexistent, although it is not long since Ireland also relied heavily on remittance income. In many developing countries today, as in Ireland previously, remittances secure the economic future of individuals, families and societies [2,36]; reduce vulnerability to economic shocks [37]; and alleviate poverty [37]. King reports the difference that remittances can make to those who remain: "Our families can only survive because we get money from abroad. The living conditions cannot be compared: those with relatives abroad live in houses, the others live in shacks" [2]. Connell and Brown echo these findings, highlighting the fact that "casual inspection of village housing enables conclusions to be quickly made on which households have migrants overseas" [24].

The choice faced by prospective migrants is stark. If no State support exists to assist households in need of housing, education and health services or to support those in need of pensions or unemployment benefit, the prospective migrant nurse, with her "internationally tradable occupation" [29] has little choice but to migrate and use her remittances to provide for her extended family. The following section takes a closer look at remittance flows and the ways in which the lives of those "back home" are altered by the money sent home by a sample of Ireland's migrant nurses.

### How much is remitted?

Many of those migrant nurses who participated in the qualitative interviews reported, in response to the question "What percentage of your income do you send?", that they were sending a considerable proportion of their salaries home in remittances:

"I send almost half, half of my salary. I just leave for my rent, a little bit for myself" (Alma).

"Oh, my income that I normally send them 80% of my income" (Ivory).

"Maybe around 70%. No, let's say around 60% because now I have to pay, I'm paying for my car ... and all the expenses in here" (Lorna).

This finding is in line with other research findings that have found that women generally send "anywhere from half to nearly all of what they earn" [38]. A United Kingdom study of migrant nurses noted that 57% remitted on a regular basis [25].

Our survey of migrant nurses painted a slightly different picture from the in-depth interviews, however, with only 23% (65) of respondents remitting more than 40% of their income, 39% (110) sending between 10% and 20% of their income and 39% (112) sending 10% or less (survey respondents who stated that they were remitters were then asked: "What proportion of your monthly salary do you send?"). Those whose children resided with them in Ireland tended to send less money home. This respondent, her first child due at the time of the interview, explains the impact of family formation on her remittance flow:

"So, I sort of a little bit prepared them already that once I have my own family, that I will cut back my remittances to them" (Francesca).

In general, respondents seemed to remit less when their living costs in Ireland began to mount, for instance as their families in Ireland expanded or as they purchased houses. Our survey of migrant nurses found that those who lived in accommodation that they owned were slightly less likely than renters to remit, with 81% (23) remitting regularly in comparison with 90% (185) of respondents who were renting accommodation:

"I don't give much to them because we have explained to them that life here's not easy as well – we are paying rent and bills very expensive as well and since I have children and you have to make sure that any problem there, you're ready, like. So they tend to understand" (Carlo).

In reaction to the high costs of living in Ireland, some respondents continued to remit at levels that caused them financial hardship in Ireland.

"Some Filipinos have pressure to send money home because ... some of their families think that they are abroad and they have lots of money" (Carlo).

Others reduced their remittance to take into account high living costs in Ireland, while expressing frustration at their inability to remit more.

"When you're here, you want to help your family as well ... your cousins, your relatives, send money for them, but if you're not able to do that, like, the satisfaction is less, I should say" (Sheela).

**Figure 2:**
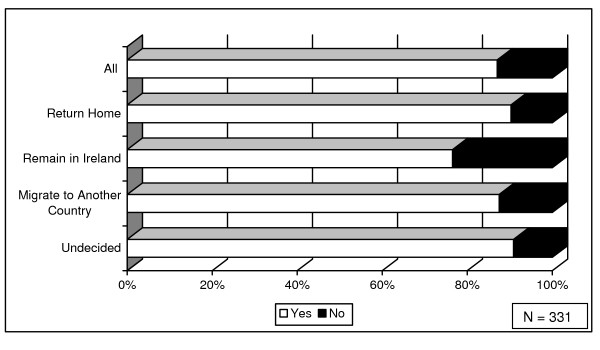
**Remittances, by future plans**.

There appeared to be a slight variation in remittance behaviour, depending on the future plans of respondents (Figure 2). For instance, among those respondents who stated that they intended to remain in Ireland, 77% (49) sent remittances home (in comparison to 87% of the wider sample). This would appear to confirm the findings of previous research that suggested that those migrants who intended to return home had a tendency to remit more generously [29], perhaps in preparation for their return.

As the survey of migrant nurses was undertaken between February and June 2009, the findings offer an insight into the impact of the economic downturn on migrant nurses and on their remittance flows. The impact of the recession had been felt in a variety of ways: all respondents had seen recent reductions to their net salary as a result of increased taxes and income levies – some targeted exclusively at public sector workers – and many found they were no longer able to supplement their incomes, due to a reduced availability of overtime and agency work. Several had also seen their spouses become unemployed. Some respondents had immediately reduced their remittance accordingly, on the basis that:

"Less overtime means less money to send back home" (226).

Other respondents found themselves unable to scale back their remittance, despite their reduced incomes in Ireland:

"Increase demand from family in Phil [Philippines] due to recession there also" (103).

"At this time my parents are sick ... I need to send money for their maintenance medication which is more than I used to send, with the levy, pay cut on the line plus no more overtime" (261).

These findings would appear to corroborate the suggestion that although remittance flows have declined as a result of the global recession, they "remain resilient compared to many other types of resource flows" [39].

Connell and Brown hypothesize that migrant nurse households are more reliable remitters because they have selected their occupation specifically in order to migrate [29] and that they are under an obligation to remit to those who funded their education. However, in conversations with migrant nurses, another possible motivation for high levels of remittances emerged. As migrants from developing countries, these nurses were acutely aware of the poverty and unmet needs that existed in their home countries. In addition to remittances to family members, some respondents also made charitable donations to their countries of origin. These charitable donations frequently involved sponsoring a student through college. As these respondents explained:

"I have two scholars ... my neighbour, because they're very poor, so I just give allowance for high school student ... And one college [student], he's almost completed. So, at least I'm helping somebody" (Vina).

"So we all give donations ... we secretly give to them ... sometimes for the child education, but sometimes they are building the house, they are in short of money, something, so we if we were work here, we give them two thousand euro. It's a big sum for them" (Elena).

Another respondent who was currently sponsoring two students through college was doing so as an indirect form of repayment to those who had sponsored her own nursing education; this represented investment in "'human capital' for the next generation" [10,24]. The reluctance among migrant workers to restrict remittance flows in line with income reductions may stem from a recognition that income reductions would have an immediate impact on the lives of family members back home. For instance, for those sponsoring students through college, disruption to the remittance flow would mean an end, or at least a pause, in their academic careers.

Unusually among migrants, most migrant health workers are employed in the public sector [40] and within the health sector, which is "expected to continue to grow at a robust pace as host societies age" [41], despite the global recession. Migrant nurses may therefore be well-placed relative to other migrant family members and might be under pressure "to send more remittance to their families, to make up for a shortfall in remittances" [39]. Regardless of reason, it would appear that the recession has left some respondent migrant nurse households "struggling and having hard times" (46).

### What a difference ... a remittance makes

Regardless of how much respondents remitted, the impact of these monies back home was felt to be significant, as these respondents explained:

"Oh, it made a great change in my life, in my family. They can eat what they want, they can do what they want, I can buy them what they want ... you gave us a good future for our family. It's really big difference. I already, I'm building now my house, which is not finished yet, but I cannot do that if I'm working in the Philippines" (Alma).

"Everything comes easier – you can have your house and that, at home, you can buy, you can have so many investments, you can send your, your children to college in a decent, proper universities and then you can help your brothers and sisters, your parents, you know what I mean, like everything. So there's a big, big change, like. So I will say the lifestyle has been changed, it was elevated" (Ivory).

**Figure 3:**
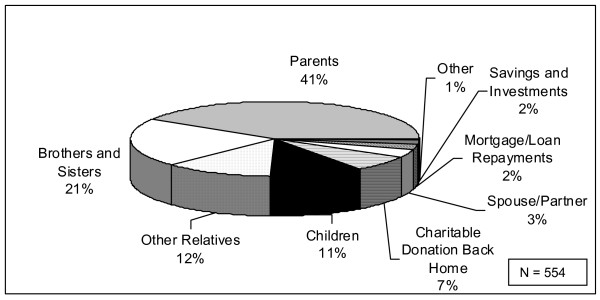
Whom do you support with remittances?

The spending patterns associated with remittances from migrant nurse respondents reflected those highlighted by other researchers [2], with remittances used to fund everything from food and daily living expenses to property and economic investments. Survey respondents were asked to indicate all those they supported via remittances (N = 554); 41% (227) supported parents, 21% (117) supported brothers and sisters and 11% (59) supported their children (Figure 3).

It would appear that the remittances from respondent migrant nurses fall into the first "wave" of remittance flows, as identified by Brown and Porine [24,42]: remittances to parents to repay their human capital investment. Far fewer respondents appeared to be directing their remittances into savings and investments or even mortgage or loan repayments back home (Figure 3).

The amount that migrant nurses could remit from Ireland was felt to compare very favourably with the amount they could save while nursing in their home countries or while working in other countries, such as Saudi Arabia:

"I was seven years back in Saudi Arabia but I have nothing. Going back home, I have nothing except for the fact that I have sent my mom for an operation and given a little bit of some gold and ... that's it, you know, at the end of the day, I have nothing in my pocket. But now, coming here now, within two years, I was able to build for my mom, a small house for her ... and, like, I could send her the money that she wanted every month and I'm still helping two of my cousins as well to go to school" (Fatima).

Remittances sent by respondent migrant nurses in Ireland enabled family members back home to pay for their health care and education expenses as well as providing support for those who were retired or unemployed. In the Irish context, such expenses would be met by the State, via the taxation system. However, in the context of developing countries, such State assistance was simply not available and remittances were necessary as a result:

" [I was] able to give some sort of a better life to my parents, both of them are retired ... they don't get any pension or anything" (Francesca).

"When I came here in Ireland, I started to send them to college and now my daughter is a nurse ... they're all in a decent, they get a decent, proper, university, proper education and a proper career" (Ivory).

"Because my eldest daughter is unemployed, so every time she needs money, I have to send her" (Lorna).

"I'm helping my brother who is at this time, always in the hospital" (Vina).

There is little doubt that the remittances sent by migrant nurses in the destination country alleviate poverty in their families in the source country. They also fund a system of support, equivalent in many respects to that which we in the developed world are accustomed to receiving from the State. In this respect, migration and the remittance flow that follows, could be considered to reduce pressure on national governments to provide welfare support services [24]. Individuals migrate and remit to provide "social protection" [37] for their families. However, this means that remittances become a necessary rather than an optional source of additional income.

### Remittance driving further migration

As remittances become necessary to enable families to meet social costs such as education, health care and pensions, pressure is placed on school leavers to select an "internationally tradable occupation" [29]: one that will enable migration and the continuation of the remittance flow. Respondents noted how remittance-related considerations shaped their own career paths; this echoes recent research findings from the United Kingdom [33]. Financial necessity, which would lead her to migrate from the Philippines, had also determined a career path for this respondent:

"I didn't want to become a nurse, for God's sake, I didn't want, that's not the kind of career that I wanted to, taking care of the patients. But at the end ... that's the job that sustains you" (Fatima).

"Being a nurse is the only, the only course, the only profession that you can really help your family with, you know, the poverty at home" (Ivory).

Nursing was considered a profession that would enable emigration, providing a "'ticket' out" [43] and therefore a career option that would ensure a remittance flow to those left behind [9]. Working overseas as a nurse was also considered to increase social standing and social status in the home country [33], perhaps even resulting in improved marriage prospects [34]. Respondents were aware that nursing salaries in their countries of origin were insufficient and that the well-being of their families depended upon their ability (and willingness) to emigrate and to remit:

"So the only way that we could alleviate as well, our own sufferings, is to come over to country as Ireland, United Kingdom and America, you know, to sustain as well, our own family" (Fatima).

"If you have one nurse at home, one nurse in the family, then you are better off because that nurse can go out of the country, can earn more, lets say double, triple the amount that we are earning at home and you can send it home and you can help the whole family" (Ivory).

However, widespread nurse migration has meant that nursing has become a career selected for its migration prospects [40]. As a result, newly trained nurses in countries such as the Philippines seek only short-term employment locally prior to their migration. Because their intention is to obtain sufficient nursing experience to facilitate their migration, these newly trained nurses "are willing to accept substandard wages – thus leading to a feedback system which works simultaneously to depress nurse wages and which encourages migration of nurses at the earliest opportunity" [44]. Nursing becomes an occupation that offers poor conditions locally, leaving early-career nurses with little choice but to migrate.

Financial and practical considerations guided the career choices of these respondents. When their own education costs had been borne by other migrants (aunts, uncles, cousins and more distant "sponsors" overseas), the importance of a career with migration prospects was heightened:

"Where the family makes a conscious decision to invest in human capital for 'export', there will be a stronger obligation for the eventual migrant to repay the family 'loan' and to participate in financing the next generation's human capital." [29].

Just as the driving force behind migration is "to support family members and support their futures at home" [10], career choice was heavily influenced by the need to remit. The increasing privatization of nurse education [30] may also influence the decision to migrate, as graduates "seek overseas employment as soon as they gain the basic clinical experience" [3], perhaps to enable them to repay tuition debts. Indeed, in countries like the Philippines, where nurse education is primarily provided by the private sector, the expectation is that these expenses will be recouped by working overseas [43]. In relation to that, our survey of migrant nurses revealed that 71% (240) of respondents received no state funding for their nursing education.

### Risks to remittance flows

The onset of recession in Ireland has implications for migrant nurses and their ability to remit, an issue frequently mentioned by those surveyed in early 2009, who saw the recently imposed tax increases and income levies in Ireland as a direct threat to their remittance. The onset of recession appears to have caused respondent migrant nurses to worry about the stability of their employment and implications for their remittance flow:

"I'm scared about the stability of my job which is affecting the quality of my life and my family back home" (169).

The sharp downturn in the Irish economy had caused respondents to feel insecure in relation to their immigration status. There was a sense that, as migrants, they were particularly vulnerable during a recession:

"I started to ask myself about my stability to live and work in this country" (52).

"As a foreigner we might be the first persons to be considered for redundancies. ... I don't have the feeling of being secure at these present times" (44).

Sometimes colleagues from the national population contributed to these concerns:

"Other Irish staff made us feel that they don't need migrant nurses any more and that we should start looking for another job because there's no more job and future for us here in Ireland" (213).

"It makes the Irish people think more 'racism' (because they think economic downturn is because of overseas people). We can feel that tension in the workplace more nowadays" (138).

The impact of the recession has been felt sharply by respondent migrant nurses. Some of those impacts are shared with the national population – for instance, the income reductions that have resulted from increased taxation and the fears arising from increased unemployment and general economic uncertainty. However, as migrants, respondents faced a range of additional concerns that have been exacerbated by the recession. They feared for their jobs, even though 80% (N = 268) hold permanent contracts. They feared that migrants will be the first to be made redundant. They feared for their immigration status and for changes in the law that might yet force them to leave:

"We do not know our future here in Ireland. We are not stable. Irish laws change very quickly. ...We are afraid" (40).

Each of these issues is given careful consideration, for they pose a potential risk to the remittance flow at a time when those at the receiving end can least afford it. Stability and security are important considerations for migrant nurses (Humphries, Brugha, McGee: 'I won't be staying here for long': A qualitative study on the retention of migrant nurses in Ireland, submitted), as is the uninterrupted flow of remittances back home. Unlike most other occupations, nursing continues to be an in-demand profession globally; overseas recruiters are targeting Irish-based nurses (ibid.), hoping to attract them to countries such as Australia and Canada. It remains to be seen whether the recession, along with wider dissatisfactions (ibid.), will motivate migrant nurses to move from Ireland:

"Once recession sets in, the economy is down ... there will be job losses, company losses and people will be dissatisfied and will look for a more greener pastures" (36).

An indication of emigration intentions of migrant nurses in Ireland can be gathered from the verification statistics of the Irish Nursing Board. Verifications are sought when a nurse, registered with the Irish Nursing Board, seeks to work in another country, such as Australia or Canada, and the Nursing Board of that country seeks to verify his or her Irish registration [23]. In 2008, verifications were sought on behalf of more than 2146 Indian and Filipino nurses in Ireland, up from 518 in 2007 (Irish Nursing Board, unpublished data). These statistics would indicate that an increasing number of migrant nurses are considering their options in terms of emigration. Despite the recession, the loss of nurses on such a scale could have serious implications for the Irish health system, particularly in light of recent health workforce projections, which indicate that "domestic supply is still expected to fall short of the recruitment requirement" [45].

## Conclusion

Remittances are more than mere financial transactions [7]. For migrant nurse respondents, remittances are a way to support their family members, ensure their continued access to health care and education and provide them with financial support in lieu of pensions or unemployment benefits. Just as migration reduces pressures on national governments to provide employment opportunities for its citizens, remittance flows serve as a source of welfare support for many citizens of the developing world. However, this means that the migrants, rather than the State, assume responsibility for ensuring continued access to social services by their family members. As a result, migration and remittances become a necessary means of ensuring the welfare of those family members unable to migrate. This "system" of welfare provision leaves those without family members overseas in a particularly perilous position. It also places an undue amount of pressure on the individual migrant to ensure the continued flow of remittances, particularly in the context of a global economic recession.

Although the common assumption has been that developing countries fund the training of health workers who subsequently migrate to the developed world, our survey reveals that 71% (240) of respondents received no State funding for their nursing education. The costs were borne privately. Such an investment is not without risk for nurses and their families, as the dividend of their investment – in the form of remittances from the migrant nurse – result only from a successful emigration.

Migrant nurses in Ireland have sacrificed (and continue to sacrifice) to ensure the continuation of their remittance flow, putting career and education plans on hold and curtailing their own household spending. Our survey of migrant nurses in early 2009 revealed a population struggling to meet their financial obligations in Ireland and back home. Increased taxes and the reduced availability of overtime have hit migrant nurses hard and yet their financial obligations are unchanged – in that they must continue to meet their financial obligations in Ireland, such as mortgage or rental payments and utility bills, while also maintaining their support of family members back home. Their obligation to those back home is as much a moral as a financial obligation and is not easily curtailed.

There is much at stake for migrant nurses and their families in the current economic climate. Their fears, in terms of the instability of employment or immigration status, are compounded by the knowledge that the welfare of their extended family depends upon their continued ability to earn and remit. How migrant nurses in Ireland will square this particular circle is difficult to say, but there is little doubt that, for the time being at least, they will continue to struggle under the double burden of increasing taxation levels in Ireland alongside the consistent (and increasing) need for their remittance back home.

## Competing interests

The authors declare that they have no competing interests.

## Authors' contributions

NH carried out the data collection and data analysis and prepared the first draft and subsequent redrafts of the paper. RB wrote the proposal. NH, RB and HMG designed the study and RB and HMG provided editorial comment on the draft paper. All authors have read and approved the final manuscript.
